# The Consumer Quality Index Hip Knee Questionnaire measuring patients' experiences with quality of care after a total hip or knee arthroplasty

**DOI:** 10.1186/1472-6963-7-60

**Published:** 2007-04-26

**Authors:** Janine H Stubbe, Tanya Gelsema, Diana MJ Delnoij

**Affiliations:** 1 NIVEL (Netherlands Institute for Health Services Research), P.O. Box 1568, 3500 BN Utrecht, The Netherlands

## Abstract

**Background:**

The Dutch Consumer Quality Index Hip Knee Questionnaire (CQI Hip Knee) was used to assess patients' experiences with and evaluations of quality of care after a total hip (THA) or total knee arthroplasty (TKA). The aim of this study is to evaluate the construct validity and internal consistency reliability of this new instrument and to assess its ability to measure differences in quality of care between hospitals.

**Methods:**

Survey data of 1,675 subjects who underwent a THA or TKA were used to evaluate the psychometric properties. Exploratory factor analyses were performed and item-total correlations and inter-factor correlations were calculated to assess the construct validity of the instrument. Reliability analyses included tests of internal consistency (Cronbach's alpha coefficients). Finally, multilevel analyses were performed to assess the ability of the instrument to discriminate between hospitals in quality of care.

**Results:**

Exploratory factor analyses indicated that the survey consisted of 21 items measuring five aspects of care (i.e. communication with nurses, communication with doctors, communication with general practitioner, communication about new medication, and pain control). Cronbach's alpha coefficients ranged from 0.76 to 0.90 indicating good internal consistency. The survey's ability to discriminate between hospitals was partly supported by multilevel analysis. Two scales (i.e. communication with nurses and communication with doctors) were able to measure differences between hospitals with respect to patients' experiences with quality of care. Logistic multilevel analyses indicated that hospitals explained part of the variation between patients in receiving information.

**Conclusion:**

These findings suggest that the CQI Hip Knee is reliable and valid for use in Dutch health care. Health care providers or health plans can use this survey to measure patients' experiences with hospital care and to identify variations in care between hospitals.

## Background

In February 2005, a hospital payment system was introduced in the Netherlands based on 'diagnosis treatment combinations' (DBCs). Prices for reimbursement are either based on fixed tariffs ('list A') or are subject to negotiations between health insurers and hospitals ('list B') [1]. Total hip (THA) and total knee arthroplasty (TKA) are procedures on 'list B' for which individual consumers and insurance companies can 'shop' among several providers. Transparent information about cost and quality of care is a prerequisite for choice, independent of whether the choice is made by individual consumers or purchasing agencies such as insurers. Individual consumers will use consumers' information to choose between different providers and health insurers should contract providers on behalf of their enrollers. Therefore, there is a growing interest in utilizing survey data to identify high quality care providers [2-4].

Work on the definition and measurement of health care quality has resulted in the availability of a range of quality indicators [5]. Two widely used indicators measuring different aspects are 'frequency and importance of experienced events' and 'degree of satisfaction with these experiences'. However, questions that ask for 'reports about events' that did or did not happen during clinical encounter, rather than a satisfaction rating tend to reflect better the quality of care and are more interpretable and actionable for quality improvement purposes [6]. Examples of standardized surveys using these report-type questions can be found in two 'families' of surveys. One of these two families is called the QUOTE (i.e. **QU**ality **O**f care **T**hrough the patients' **E**yes) family [7-13], which was developed in the Netherlands to measure patients' experiences with quality of care and to assess the importance consumers attach to the different quality aspects of care. Apart from the generic items that each questionnaire comprises, in addition group-specific, care-specific, or disease-specific items are included in the questionnaire. However, one of the disadvantages of the questionnaire is that it uses answering categories that are internationally not widely used: no, not really, on the whole yes, and yes.

The second family of surveys is the Consumer Assessment of Healthcare Providers and Systems (CAHPS^®^), which is widely used in the USA and well-established [14-20]. These surveys are designed to collect data that will enable consumers to compare patients' perspectives on the quality of care. The answering categories of the CAHPS questionnaires affiliate well to international research, however, they do not take the importance of quality aspects into account, and do not include group-, care- or disease-specific items.

To be able to measure both performance and importance of quality aspects on widely used scales, both families of surveys were combined to create a new family of surveys in the Netherlands, which was called the Consumer Quality Index (CQI). Considering the comparable surgery and recovering stage for knee and hip replacements, it was decided to develop one questionnaire measuring patients' evaluation with quality of care after a THA or TKA, which was called the Consumer Quality Hip Knee Questionnaire, abbreviated as the CQI Hip Knee. The development of the CQI Hip Knee has been described in detail elsewhere [21].

The aim of this article is two-folded. First of all, we want to evaluate the construct validity and reliability of the Dutch CQI Hip Knee. Secondly, we want to assess its ability to measure differences in patients' experiences with quality of care between different hospitals. With the latter, it is important to take into account patients' characteristics that are not randomly distributed across hospitals, because these characteristics may cause differences in experiences with quality of care between hospitals. Characteristics of patients have previously been studied to assess their impact on levels of patient satisfaction and experiences with quality of care [22-26]. Arah and colleagues [22] evaluated the association between respondent characteristics and three global ratings (of nurse, doctor, and hospital), using multiple linear regression models. Age and general health status consistently appeared to contribute to differences in between-hospital variations in patient experiences and ratings. Therefore, we correct for different individual characteristics when comparing patients' experiences with quality of care between hospitals.

Two research questions were derived from the aims of this article: "*Is the CQI Hip Knee reliable and what is the dimensional structure of the instrument?*", and "*Does the CQI Hip Knee measure differences between hospitals in patients' experiences with quality of care after a hip or knee operation?*".

## Methods

### The CQI Hip Knee questionnaire

The development of the Dutch CQI Hip Knee was based on three different sources. To get a clear picture of those specific quality aspects that are important to patients who underwent a THA or TKA, focus group interviews were conducted by two researchers. A total of 31 patients from the department of orthopaedic surgery from two different hospitals were recruited to participate in the focus groups. These patients underwent a total knee or hip replacement within the last three months. Considering the comparable surgery and recovering stage for knee and hip replacements, it was decided to combine the two patient groups in one focus group. The interviews lasted two hours and were audio taped. To structure the interview, quality of care was divided into three topics: pre-hospital care, care in the hospital, and follow-up care. For each period, two questions were asked to serve as a starting-point: "*Which aspects of the quality of (pre-/follow-up) care regarding your hip or knee operation do you find important*?", and "*What are your experiences with the quality of (pre-/follow-up) care regarding your hip or knee operation*?". The taped interviews were completely transcribed and analysed by hand. Statements about similar themes were categorized. The pool of items generated by the focus group interviews was evaluated by nine professionals (i.e. doctors, nurses, and researchers).

Secondly, the Dutch H-CAHPS measuring patients' experiences with quality of hospital care was used to generate items [22]. This questionnaire is part of the CAHPS family [14-20], and was shown to be a reliable, valid and feasible instrument for assessing the quality of hospital care from the patients' views [22]. Answering categories are based on a four-point Likert scale ranging from never to always, or based on a three-point scale: not a problem, a small problem, and a big problem. We selected the items measuring quality of hospital care from a patient perspective.

Thirdly, we used the QUOTE family [7-13] to generate items. These questionnaires conceptualize patients' experiences with quality of care in two dimensions: performance and importance [27]. Performance refers to the actual experience of patients with the quality aspects, and importance relates to the fact that people see some quality aspects as more significant than others. They reflect what people see as desired qualities in health care. Relevant items were selected from the QUOTE-Rheumatic-Patients instrument [12], the QUOTE-Cataract [8,9], and the QUOTE-diabetes questionnaire which is still in progress, and adjusted for hip and knee patients.

Focus group interviews, and selecting items from the H-CAHPS and the QUOTE-family resulted in the CQI Hip Knee. The exact selection of items from each of the three sources has been described in detail elsewhere [28]. The CQI Hip Knee consists of two questionnaires, i.e. the CQI Hip Knee Experience and the CQI Hip Knee Importance. The CQI Hip Knee Experience contains general items (e.g. age, education, ethnicity, and patient's health), four global ratings (of doctors, nurses, general practitioner and hospital), and 60 performance items referring to the actual experience of patients with the quality aspects. The answering formats of the performance items are: 1) never, sometimes, often, and always, or 2) not a problem, a small problem, and a big problem. The global ratings range from 0 to 10, with a score of 10 indicating the best possible score.

The CQI Hip Knee Importance also comprises demographical items, and in addition consists of importance items asking how important hip and knee patients value the quality aspects of the CQI Hip Knee Experience with answering categories ranging from not important to very important. The outcome of the CQI Hip Knee is valuable, because it shows which quality aspects patients find important and how they evaluate these aspects.

### Subjects

Case finding was done through the administration of four big insurance companies. The Dutch CQI Hip Knee was sent to 2,456 patients for whom costs of a knee/hip operation were claimed within the last three months. At the end of the data collection, 1,929 patients from 43 different hospitals had returned the questionnaire. Of these patients, 72 respondents were not willing or able to participate. Patients were not included into the analyses if they responded negative to the question whether or not they underwent a total hip or knee operation (n = 14) or if this information was missing (n = 125). Furthermore, patients who stated that they did not answer the questions themselves (n = 34) or who filled in less than half of the core items (n = 9) were also excluded from the analyses. Therefore, a total of 254 subjects were excluded from the analyses, resulting in a sample of 1,675 patients (response rate = 68,2%).

For the comparisons between hospitals, individual characteristics were taken into account. We therefore excluded respondents with missing values on age (N = 15), gender (N = 12), education (N = 86), self-reported physical health status (N = 36), and self-reported mental health status (N = 18). In total, 167 respondents were excluded from the multilevel analyses, resulting in 1,508 respondents from 43 different hospitals. The mean number of patients per hospital was 40, with a minimum of 13 and a maximum of 121 patients. The 167 respondents did not significantly differ from the remaining 1,508 respondents on the four global ratings (of doctor, nurses, general practitioner and hospital).

### Analytic approach

In this paper we focus on patients' experiences with quality of hospital care and therefore, we only used the CQI Hip Knee Experience and selected the 60 items measuring quality aspects of hospital care. To evaluate the construct validity of this questionnaire, an exploratory factor analysis was conducted and item-total correlations correcting for item overlap were calculated. When variables are measured on a nominal (yes/no) scale, linear factor analysis (e.g. common factor analysis [29]) may yield biased estimates of the factor structure [30,31]. Therefore, we did not include 29 nominal items measuring quality aspects. Participants responded to the remaining 31 items by stating how often they experienced a certain situation during their THA/TKA episode with scores ranging from one (never) to four (always). The higher the score on the domain, the higher the patient's perception of quality of care. A total mean score for each subscale was computed by summing the responses to the items (five items were reverse-coded) and dividing these sum scores by the number of items filled in. At least half of the subscale items had to be filled in, otherwise patients were excluded from the analyses.

We performed the exploratory factor analysis (EFA) with a direct oblimin rotation. We preferred this oblique rotation to an orthogonal rotation (i.e. varimax), because several factors might be interrelated. An oblique rotation could also result in independent factors if that provides a better fit. The number of factors was determined by Kaiser's criterion [32]. However, this method sometimes retains too many factors, and therefore we also used the scree test proposed by Cattell [33]. In general, factor loadings are considered meaningful when they exceed 0.30 or 0.40 [29]. Therefore, items were only assigned to a factor if the magnitude of their factor loading exceeded 0.40. To evaluate the construct validity the item-total correlations (ITC) correcting for item overlap were calculated [34]. Nunnally and Bernstein [35] proposed that these correlations should be greater than 0.40. Inter-factor correlations were calculated to give further insights into the interpretability of the constructed factors as separate scales. Correlations of less than 0.70 support the multidimensionality of the questionnaire [36].

Secondly, to evaluate the reliability of the questionnaire, Cronbach's alpha coefficients of the different scales were calculated [37]. The commonly used rule of thumb is that an alpha exceeding the value of 0.70 indicates a satisfactory internal consistency for a scale [35]. Furthermore, we analyzed whether the presence or removal of any of the items would increase the internal consistency of the scale. Nine items of the 29 nominal items were about information. We calculated a Cronbach's alpha coefficient for these nine dichotomous information items to explore whether these nine items form one scale. In SPSS, the Cronbach's alpha will produce the same result as the Kuder-Richardson Formula 20 (K-R-20), which is the alternative method to assess the internal consistency for dichotomous items [38]. A high KR-20 coefficient (e.g., >0.90) indicates a homogeneous test.

Thirdly, to assess the questionnaire's ability to measure differences in quality of care between hospitals, multilevel analyses were performed using the MLwiN software package [39]. Four separate multilevel analyses were carried out on the following domains of the CQI Hip Knee Experience: communication with nurses, communication with doctors, communication about new medication, and pain control. Number of patients was relatively small on the domain communication about new medication, because patients only filled in these questions if new medication was prescribed. Because experience with quality of hospital care is independent of the general practitioner, we did not take the dimension about communication with general practitioner into account in the multilevel analyses. Furthermore, we performed multilevel analyses on the global hospital rating, because we hypothesised that these ratings may vary between hospitals.

Multilevel analyses deals with data that are hierarchically structured [40] with one dependent variable measured at the lowest level and exploratory variables at the same and higher levels. In the present study, we assume that the experiences with quality of hospital care of patients are depended on the hospital in which they were operated. Our dataset had two levels: individual patients (level 1) nested within hospitals (level 2). It is expected that patients who are operated at the same hospital should agree more on their evaluations of hospital care than patients who are operated at different hospitals. Therefore, the within-hospital variation should be significantly smaller than the between-hospital variation. The intra-class correlation is an index of the ratio between-to-within hospital variation [41]. Variance can only be positive and therefore the value of the ICC ranges between 0 and 1. An ICC of zero suggests that patients' experience with quality of care is not related to the hospital in which they were operated.

Two different, nested models are used to fit the data. First, we fitted a random intercept model in which the variance of the dependent variable is partitioned into variance that can be attributed at the individual level, and at the hospital level (Model 1), i.e. this model does not include any explanatory variables but only estimates the overall mean (intercept) and the hospital and individual differences in the dependent variable. Secondly, in model 2, individual characteristics (age, gender, education, self-rated physical health, and self-rated mental health) were entered into the equation and the amount of variance explained by these variables at the individual level was estimated. The proportional change in variance (PCV) was calculated to evaluate how much of the variance in the first model is attributable to differences in individual characteristics [42]. Regression coefficients were estimated to get insight in the contribution of each characteristic. This last model is relevant, because one of the goals of the questionnaire is to understand individual variations within hospitals. Therefore, it is important to investigate whether survey results might be influenced by factors that are not distributed randomly across hospitals, and if so, to adjust for differences in patient mix when making comparisons between hospitals.

Although, we did not take the dichotomous variables into account in the factor analyses, they may be able to measure differences between hospitals in patients' experiences with quality of care. Of the dichotomous quality aspects, we selected one dichotomous information item which was rated by patients as most important according to the CQI Hip Knee Importance and performed a logistic multilevel analysis. This item asked patients if they received any information about symptoms or health problems to which they had to pay attention to after they were discharged from the hospital. As with the previous multilevel analyses, first the random-intercept model was fitted to the data, followed by the model in which the individual characteristics were taken into account. Logistic multilevel analysis does not estimate regression coefficients, but calculates the odds ratios (OR). Furthermore, ρ is calculated, which can be interpreted as the ICC in linear multilevel analyses.

## Results

Table 1 summarizes the respondents' characteristics and the mean global hospital rating. A high mean rating of 8.4 was given for hospitals. No significant difference in mean global hospital rating between THA and TKA patients were found (p = 0.16).

**Table 1:** Individual characteristics and mean scores for the global ratings of hospital of the 1,508 respondents.

Characteristics	% or mean (SD)
Age (years)	
18–64 (%)	29.4
65+ (%)	70.6
	
Gender	
Male (%)	27.9
Female (%)	72.1
	
Education	
No or less than secondary education (%)	28.2
Secondary or higher education (%)	71.8
	
Self-reported physical health	
High (%)	86.3
Low (%)	13.7
	
Self-reported psychological health	
High (%)	70.4
Low (%)	29.6
	
Global rating (ranging from 0 to 10)	
Mean hospital rating (SD)	8.4 (1.4)

SD, standard deviation

Two exploratory factor analyses were conducted on data of the 1,675 patients who underwent a THA or TKA. The first factor analysis resulted in eight factors having eigenvalues greater than 1 (9.95, 2.31, 1.95, 1.71, 1.48, 1.17, 1.09, 1.02), and thus satisfied Kaiser's criterion. The amount of variance explained by the eight factors was 66.7%

Table 2 gives an overview of the first exploratory factor analysis. Factor loadings are considered meaningful when they exceed 0.30 or 0.40. Our results showed that that three of the 31 core items (Q7, Q11, and Q 12) were unrelated to any of the eight factors. However, we decided to include these items into the second factor analysis, because their loadings were very close to 0.30 or to 0.40 and were salient in the expected factor. No items had loadings exceeding 0.40 on two different factors.

**Table 2:** Factor loadings of the 31 items according to the first exploratory factor analysis with oblimin rotation. Cronbach's alpha whole scale (α1), Cronbach's alpha of scale if item was deleted (α2), and item total correlation correcting for overlap (ITC) are displayed.

**nr**	**Item description**	**loading**	**α1**	**α2**	**ITC**
	*Factor 1: Communication with nurses*		0.90		
Q4	Nurses treat me with respect	0.68		0.89	0.69
Q5	Nurses take me seriously	0.50		0.88	0.72
Q6	Nurses listened carefully	0.52		0.88	0.77
Q7	Nurses explained things clearly	0.29		0.89	0.61
Q8	Nurses spent enough time	0.51		0.88	0.74
Q9	Help as soon as you wanted	0.53		0.89	0.64
Q12	Nurses kept their appointments	0.38		0.89	0.65
Q15	Help with bathing as soon as you wanted	0.73		0.89	0.60
Q17	Help with going to the toilet as soon as you wanted	0.93		0.89	0.60

	*Factor 2: Communication with doctors*		0.82		
Q20	Doctors treat me with respect	0.86		0.79	0.64
Q21	Doctors take me seriously	0.81		0.78	0.72
Q22	Doctors listened carefully	0.80		0.78	0.72
Q23	Doctors explained things clearly	0.63		0.80	0.56
Q24	Doctors spent enough time	0.72		0.78	0.68
Q27	Doctors kept their appointments	0.74		0.80	0.54

	*Factor 3: Communication with general practitioner*		0.90		
Q67	General practitioner gave good advice	0.94		n.c.	0.82
Q68	General practitioner let me decide about treatment	0.95		n.c.	0.82

	*Factor 4: Asking things more than once*		0.57		
Q10	Nurses asked same thing more than once	0.87		n.c.	0.46
Q25	Doctors asked same thing more than once	0.85		n.c.	0.42

	*Factor 5: Communication about new medication*		0.59		
Q35	Patients in your room support you	0.77		0.76	0.20
Q59	Told what new medicine was for	0.65		0.32	0.53
Q60	Told side-effects of new medicine	0.66		0.29	0.53

	*Factor 6: Environment hospital*		0.63		
Q11	Nurses gave conflicting information	0.39		0.61	0.30
Q31	Temperature room was pleasant	0.45		0.57	0.39
Q33	Environment room quiet at night	0.63		0.56	0.41
Q34	(Visitors of) patients in your room burden on you	0.79		0.60	0.31
Q38	Enough privacy	0.43		0.57	0.37

	*Factor 7: Pain control*		0.76		
Q56	Pain controlled	0.83		n.c.	0.62
Q57	Everything done to help with pain	0.70		n.c.	0.62

	*Factor 8: Rest items*		0.11		
Q26	Doctors gave conflicting information	-0.54		n.c.	0.06
Q32	Bathroom clean	0.65		n.c.	0.06

n.c. = not calculated. With two items in one scale, Cronbach's alpha of scale if item is deleted (α2) can not be calculated.

Internal consistency was investigated by calculating Cronbach's alpha coefficients. The fourth column (α1) of Table 2 shows that the coefficients ranged from 0.11 to 0.90. Four factors had poor reliability resulting in Cronbach's alpha coefficients of 0.11, 0.57, 0.59, and 0.63. Removal of any of the items did not increase the alpha coefficient to the threshold of 0.70 (see column 5 of Table 2), and therefore we were not able to construct reliable scales with these items. However, removing Q35 from factor 5 increased Cronbach's alpha coefficient up to 0.76. We therefore only removed Q35 from this scale and did not eliminate the whole scale. In total, five reliable scales could be formed and this was in agreement with the scree test. Furthermore, the internal consistency was tested for nine dichotomous information items. KR-20 coefficient of 0.39, indicated poor reliability of this scale.

The remaining 21 items were entered in the second factor analysis to estimate the variance explained by the five factors after excluding Q35. This second factor analysis was restricted to identification of five factors explaining 68% of the total variance in patients' experiences with quality of care. Table 3 shows the results of this second factor analysis.

**Table 3:** Factor loadings of the 21 items according to the second factor analysis with oblimin rotation. Cronbach's alpha whole scale (α1), Cronbach's alpha of scale if item was deleted (α2), and item total correlation correcting for overlap (ITC) are displayed. Factor loadings exceeding 0.40 are shown.

**nr**	**Item description**	**loading**	**α1**	**α2**	**ITC**
	*Factor 1: Communication with nurses*		0.90		
Q4	Nurses treat me with respect	0.78		0.89	0.69
Q5	Nurses take me seriously	0.75		0.88	0.72
Q6	Nurses listened carefully	0.76		0.88	0.77
Q7	Nurses explained things clearly	0.43		0.89	0.61
Q8	Nurses spent enough time	0.76		0.88	0.74
Q9	Help as soon as wanted	0.73		0.89	0.64
Q12	Nurses kept their appointments	0.67		0.89	0.65
Q15	Help with bathing as soon as you wanted	0.68		0.89	0.60
Q17	Help with going to the toilet as soon as you wanted	0.79		0.89	0.60

	*Factor 2: Communication with doctors*		0.86		
Q20	Doctors treat me with respect	-0.86		0.84	0.65
Q21	Doctors take me seriously	-0.85		0.83	0.72
Q22	Doctors listened carefully	-0.82		0.82	0.75
Q23	Doctors explained things clearly	-0.57		0.85	0.59
Q24	Doctors spent enough time	-0.69		0.83	0.69
Q27	Doctors kept their appointments	-0.64		0.85	0.55

	*Factor 3: Communication with general practitioner*		0.90		
Q67	General practitioner gave good advice	0.95		n.c.	0.82
Q68	General practitioner let me decide about treatment	0.95		n.c.	0.82

	*Factor 4: Communication about new medication*		0.76		
Q59	Told what new medicine was for	0.79		n.c.	0.61
Q60	Told side-effects of new medicine	0.81		n.c.	0.61

	*Factor 5: Pain control*		0.76		
Q56	Pain controlled	0.85		n.c.	0.62
Q57	Everything done to help with pain	0.66		n.c.	0.62

n.c. = not calculated. With two items in one scale, Cronbach's alpha of scale if item is deleted (α2) can not be calculated.

The five factors measuring five aspects of THA/TKA care are communication with nurses, communication with doctors, communication with general practitioner, communication about new medication, and pain control. Factor loadings all exceeded 0.40 including the factor loading of Q7, Q11, and Q12, which confirmed the choice of retaining these items in the second factor analysis. Cronbach's alpha coefficients ranged from 0.76 to 0.90 indicating good reliability of the five factors. All item-total correlations corrected for overlap were higher than the threshold of 0.40, indicating good construct validity. Table 4 displays the inter-factor correlations. All correlations were less than 0.70 supporting the multidimensionality of the questionnaire, i.e. the scales could be read as separate scales. No significant difference between THA and TKA patients were found for the five domains (p > 0.05).

**Table 4:** Inter-factor correlations for the five subscales.

	Factor 1	Factor 2	Factor 3	Factor 4	Factor 5
Factor 1	1.00				
Factor 2	0.42	1.00			
Factor 3	-0.49	-0.14	1.00		
Factor 4	0.39	0.12	-0.32	1.00	
Factor 5	0.39	0.03	-0.33	0.23	1.00

Factor 1, communication with nurses; Factor 2, communication with doctors; Factor 3, communication with general practitioner, Factor 4, communication about new medication; Factor 5, pain control.

Multilevel analyses were performed to assess the ability of the questionnaire to measure differences in quality of care. Table 5 shows the results of these analyses. Intercepts of model 1 represent the overall mean scores of patients on the five outcome variables. These scores can range from one to four for the domain variables and from zero to ten for the hospital rating, with high scores reflecting positive experiences of patients with quality of care. For three of the four domain variables, the mean scores were close to four, indicating that patients had positive experiences with care. This was in agreement with the high hospital rating of 8.4.

**Table 5:** Model fitting results of the multilevel analyses for the domains communication with doctor, communication with nurses, communication about new medication, and pain control, and for the global rating of hospitals (standard errors added in parentheses).

**Outcome variable**	**intercept**	**β age^1^**	**β gender^1^**	**β education^1^**	**β physical health^1^**	**β mental health^1^**	**Var patients**	**Var hospital**	**Var tot^2^**	**PCV^3^**	**ICC^4^**
*Doctor (N = 1469)*											
Model 1	3.523 (0.022)*	-	-	-	-	-	0.290 (0.011)*	0.011 (0.005)*	0.301	reference	0.04
Model 2	3.632 (0.048)*	-0.021 (0.032)	-0.059 (0.031)	0.021 (0.032)	-0.139 (0.045)*	-0.152 (0.034)*	0.279 (0.010)*	0.010 (0.004)*	0.289	4.2%	0.03
											
*Nurses (N = 1507)*											
Model 1	3.508 (0.018)*	-	-	-	-	-	0.205 (0.008)*	0.007 (0.003)*	0.212	reference	0.03
Model 2	3.675 (0.038)*	-0.031 (0.026)	-0.081 (0.026)*	-0.024 (0.026)	-0.148 (0.037)*	-0.158 (0.028)*	0.193 (0.007)*	0.005 (0.002)*	0.198	7.1%	0.03
											
*Medication (N = 630)*											
Model 1	2.896 (0.043)*	-	-	-	-	-	1.142 (0.064)*	0.000 (0.000)	1.142	reference	n.c.
Model 2	3.082 (0.139)*	-0.169 (0.090)	-0.163 (0.094)	0.187 (0.100)	-0.331 (0.133)*	-0.171 (0.101)	1.087 (0.061)*	0.000 (0.000)	1.087	5.1%	n.c.
											
*Pain control (N = 1484)*											
Model 1	3.594 (0.018)*	-	-	-	-	-	0.357 (0.013)*	0.003 (0.003)	0.360	reference	n.c.
Model 2	3.632 (0.050)*	0.008 (0.035)	-0.023 (0.035)	0.028 (0.036)	-0.120 (0.050)*	-0.101 (0.038)*	0.352 (0.013)*	0.001 (0.002)	0.353	2.0%	n.c.
											
*Rating hospital (N = 1490)*											
Model 1	8.422 (0.046)*	-	-	-	-	-	1.818 (0.067)*	0.031 (0.018)*	1.849	reference	n.c.
Model 2	8.789 (0.113)*	0.094 (0.078)	-0.135 (0.078)	-0.228 (0.080)*	-0.265 (0.112)*	-0.440 (0.085)*	1.744 (0.065)*	0.023 (0.016)*	1.767	4.6%	n.c.

*p < 0.05

^1^Reference group age = younger than 65; reference group gender = males; reference group education = low education; reference group physical health = good physical health; reference group mental health = good mental health.

^2^Var tot (total variance) = Var patients + Var hospital

^3^PCV (proportional change in variance) = (Var tot model 1 - Var tot model 2)/(Var tot model 2) * 100%

^4^ICC (intra-class correlation) = Var hospital/(Var patients + Var hospital)

n.c. = not calculated. The variance explained by the hospital level is not significant and, therefore, the ICC is not calculated.

For all five outcome variables, Model 1 and Model 2 indicated that there was significant variation in patients' experience in quality of hospital care (column eight). Except for communication about new medication, regression coefficients (column three to seven) showed that self-reported physical health and self-reported mental health accounted for part of the variance in patients' experiences. Gender showed significant results for communication with nurses, and education accounted for part of the variation in hospital rating. Age did not significantly influence any of the five outcome variables. The proportional change in variance (PCV) ranged from 2.0% to 7.1% for the five outcome variables, indicating that 2.0% to 7.1% of the variance in the first model was attributable to differences in individual characteristics.

For two of the five outcome variables (i.e. communication with doctors, and communication with nurses), variation at hospital level was significant (column nine and ten). There was no significant variation at the hospital level for the domains communication about new medication, pain control, and global hospital rating. After inclusion of the individual characteristics age, gender, education, physical health, and mental health, the amount of variation explained by hospital decreased a little, however, it still remained significant for the two outcome variables communication with doctors and communication with nurses.

Of the nine dichotomous quality aspects, the item "After you were discharged, did you receive information about the symptoms or health problems you had to pay attention to?" was rated as most important by patients according to the CQI Hip Knee Importance. The results of the logistic multilevel analyses are shown in Table 6. The probability of receiving information was 0.40 for model 1. Furthermore, μ_0j _showed that there was variation between hospitals in giving information to patients. Age, education, physical and mental health were significantly different from 1.0.

**Table 6:** Model fitting results of the logistic multilevel analysis for the dichotomous variable "After you were discharged, did you receive information about the symptoms or health problems you had to pay attention to?".

**Outcome variable (N = 1,456)**	**Model 1**	**Model 2**
Probability (π) (95% CI)	0.40 (0.38; 0.42)	0.21 (0.18; 0.25)
OR age (18–65 = reference) (95% CI)	-	1.66 (1.29; 2.13)
OR sex (males = reference) (95% CI)	-	1.21 (0.95; 1.55)
OR education (low education = reference) (95% CI)	-	1.39 (1.08; 1.79)
OR physical health (high = reference) (95% CI)	-	1.60 (1.14; 2.25)
OR mental health (high = reference) (95% CI)	-	1.40 (1.08; 1.82)
Variance hospitals (SE)	0.12 (0.06)*	0.11 (0.05)*
Variance patients (SE)	0.99 (0.04)^1^	0.99 (0.04)^1^
P	0.04	0.03

*p < 0.05

^1^By definition, individual level variance is one if the binomial distribution holds.

SE, standard error

95% CI, 95% confidence interval

## Discussion

In this article, we investigated the psychometric properties of the CQI Hip Knee, and evaluated its ability to discriminate between hospitals. The following five composites could be formed: communication with nurses, communication with doctors, communication with general practitioner, communication about new medication, and pain control. Psychometric properties of the questionnaire were good. Internal consistency ranged from 0.76 to 0.90 and all item-total correlations corrected for overlap were higher than the threshold of 0.40. Inter-factor correlations were less than 0.70 supporting the multidimensionality of the questionnaire. However, inter correlations between factors were not equal to zero, supporting the use of an oblique rotation (i.e. oblimin rotation). Ten items did not meet the psychometric standards. However, these items might give important information on the quality of care. Therefore, we are reluctant to eliminate these items from the questionnaire, even though they cannot be assigned to a certain domain. No significant difference in quality of care between THA and TKA patients were found (p > 0.05), which confirmed the choice to develop one questionnaire measuring patients' evaluation with quality of care after a THA or TKA.

Multilevel analyses revealed that several individual characteristics significantly influence patients' experience with quality of hospital care. Patients with low levels of self-reported physical and mental health rated their hospital lower and had less positive experiences with communication with doctor, nurses, and pain control. Self-reported physical health was also associated with less positive experiences with communication about new medication. This is in keeping with a review by Pascoe and colleagues [25], who found that patients with better health tend to be more satisfied. In addition, Arah and colleagues [22] evaluated the association between respondent characteristics and three global ratings, using multiple linear regression models. Results showed that poorer health status was significantly related to lower global ratings by surgical and medical patients. Contrary to our results, both studies showed that age was the variable having the most consistent effect, being associated with higher satisfaction with care [22,25]. We were not able to replicate this finding in our study. This might be explained by the fact that we had a relatively old and homogenous patient sample. More than 70% of the patients undergoing THA or TKA was 65 years or older. In the study by Pascoe and colleagues [25] and Arah and colleagues [22] age samples were more heterogeneous.

Furthermore, for the domain communication with doctors and communication with nurses, the hospital in which patients were operated significantly contributed to differences between patients' experiences with quality of care. Furthermore, logistic multilevel analyses revealed that the probability of receiving information was explained by the hospital in which patients were operated. Therefore, we concluded that on an item level the CQI Hip Knee is also able to measure differences between hospitals in patients' evaluation of a quality aspect of care.

In general, Dutch patients who underwent a THA or TKA have positive experiences with the quality of care. This is reflected in the high global rating and the high mean sum scores on the domains communication with doctor, communication with nurses, and pain control. These high scores might be related to our homogenous sample of older patients, which is in agreement with two studies suggesting that older patients may be more satisfied, because they are less critical, become mellow and accepting, and they feel more reluctant than younger patients to pass negative judgement on their care [23,26]. Another possibility is that older patients happened to be treated in a more thorough or responsive manner than younger patients. Two studies provide support for this possibility [43,44].

These two suggestions (i.e. response tendency and positive treatment received) might also be important in explaining little variation in patients' experiences. Our older patients had high levels of experiences with quality of hospital care and this was also reflected in the small differences between hospitals in patients' experiences with quality of care. However, these small differences are robust, and for two of the five subscales, the instrument is able to discriminate between hospitals.

These small but robust effects are reflected by all ICCs in this sample ranging from 0.03 to 0.04, indicating small differences between hospitals in patients' experience with the quality of care. However, mostly ICCs are lower, for example, the median ICC calculated for more than 1000 primary care variables was 0.01 [45].

There was no variation between hospitals in the experiences of patients' quality of care with regard to pain control. This domain consisted of the following two questions: *"During this hospital stay, how often was your pain well controlled*?", and *"During this hospital stay, how often did the hospital staff do everything they could to help you with your pain?"*. In the literature, pain is known to be very subjective. For example, a study by Tang and Gibson [46] showed that individuals with higher trait anxiety (a greater disposition to experience anxiety) tend to exacerbate perceived pain stimulations more than lower trait anxious individuals. This might explain the fact that there is variation in patients' pain experiences regardless of the hospital carrying out the operation.

One of the limitations of this study is that we were not able to include hospital information at the second level, because we did not have this information. It might be very interesting to investigate this in the future, for it can be used by Dutch health plans in their negotiations with contracted health providers and provide information about best practices.

## Conclusion

It is possible to reliably and meaningfully measure patients' experience with quality of care after a THA or TKA. Use of the Dutch CQI Hip Knee questionnaire allows valid comparisons between hospitals on two domains (i.e. communication with doctors and communication with nurses), and on one item level.

## Competing interests

The author(s) declare that they have no competing interests.

## Authors' contributions

JS performed the psychometric and multilevel analyses and was the principal author of the article. TG constructed the questionnaire, collected the data and performed the qualitative analyses. DD designed the study, supervised the project and assisted in writing the final draft of the article. All authors read and approved the final manuscript.

## Pre-publication history

The pre-publication history for this paper can be accessed here:


